# Robust and Real-Time Detection and Tracking of Moving Objects with Minimum 2D LiDAR Information to Advance Autonomous Cargo Handling in Ports

**DOI:** 10.3390/s19010107

**Published:** 2018-12-29

**Authors:** Victor Vaquero, Ely Repiso, Alberto Sanfeliu

**Affiliations:** Institut de Robòtica i Informàtica Industrial, CSIC-UPC, Llorens i Artigas 4-6, 08028 Barcelona, Spain; sanfeliu@iri.upc.edu

**Keywords:** lidar perception, object detection, object tracking, single-layer laser scanner, DATMO, multi-hypothesis tracking, autonomous driving, autonomous transportation of cargo

## Abstract

Detecting and tracking moving objects (DATMO) is an essential component for autonomous driving and transportation. In this paper, we present a computationally low-cost and robust DATMO system which uses as input only 2D laser rangefinder (LRF) information. Due to its low requirements both in sensor needs and computation, our DATMO algorithm is meant to be used in current Autonomous Guided Vehicles (AGVs) to improve their reliability for the cargo transportation tasks at port terminals, advancing towards the next generation of fully autonomous transportation vehicles. Our method follows a Detection plus Tracking paradigm. In the detection step we exploit the minimum information of 2D-LRFs by segmenting the elements of the scene in a model-free way and performing a fast object matching to pair segmented elements from two different scans. In this way, we easily recognize dynamic objects and thus reduce consistently by between two and five times the computational burden of the adjacent tracking method. We track the final dynamic objects with an improved Multiple-Hypothesis Tracking (MHT), to which special functions for filtering, confirming, holding, and deleting targets have been included. The full system is evaluated in simulated and real scenarios producing solid results. Specifically, a simulated port environment has been developed to gather realistic data of common autonomous transportation situations such as observing an intersection, joining vehicle platoons, and perceiving overtaking maneuvers. We use different sensor configurations to demonstrate the robustness and adaptability of our approach. We additionally evaluate our system with real data collected in a port terminal the Netherlands. We show that it is able to accomplish the vehicle following task successfully, obtaining a total system recall of more than 98% while running faster than 30 Hz.

## 1. Introduction

**Figure 1:**
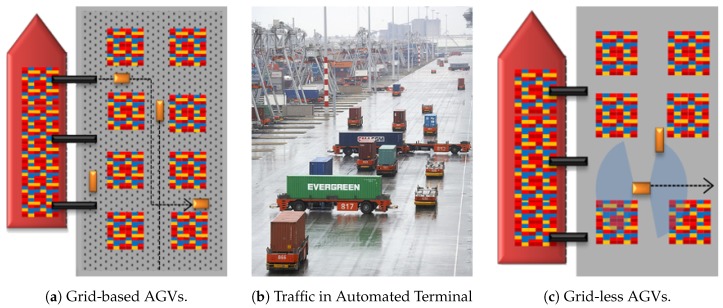
Representation of a current automated port terminal (**a**) and the aimed next generation grid-less AGVs (**c**). Vessels arrive at the seaside of the port and the cranes unload or load the containers. AGVs move the containers from the cranes area to the stacking area represented here with multi-color squares. Presently, AGVs follow pre-defined paths which limit their movements (**a**,**b**). Grid-less AGVs will provide vehicles with decision capacity and freedom of movement as seen in (**c**).

Presently, up to 90% of the international trade volume of manufactured goods is performed by means of multimodal containers [1] and, according to the United Nations Review of Maritime Transport [2], the container shipping industry is the fastest-growing segment of freight transportation. Port terminals must achieve a high level of productivity and efficiency in container throughput to handle this uprising and concentrated container traffic and meet future demands. One of the least efficient and costly processes in ports comes from internal transportation [3], as for example the observed area in Figure 1. It refers to the container movement between the harbor, where the cranes move the containers from/to the vessels, and the storage area where the containers are placed.

Automated Container Terminals (ACTs) are a good solution to this bottleneck, as it allows a reliable and continuous operation on transport processes as well as decreases possible human errors. To move the containers through the transport areas, ACTs commonly employ Automated Guided Vehicles, as seen in Figure 1b. However, container terminals are especially dynamic areas without pre-defined structures such as paths, sidewalks, buildings or signs (see Figure 1b). Moreover, containers can be stacked in diverse ways in different areas that may even fully change in short periods of time. This fact makes it especially challenging to obtain any kind of durable map or to apply standard automation techniques to help distinguishing drivable areas or even static obstacles. The current generation of AGVs therefore move attached to electro-magnetic grids embedded in the surface of transport areas, as can be observed in Figure 1a,b. This technology requires a high investment and limits AGV usability to only correctly equipped areas of ports and terminals. In addition, every movement is totally pre-defined by centralized software that regulates the position, direction and speed of each vehicle, which is a limitation that may cause problems such as collisions or deadlocks [4].

This complex scenario sets our motivation to advance towards the next generation of fully aware AGVs. We aim to build a DATMO system to provide current grid-based AGVs with wider autonomy and capabilities to explore and move through the whole port transport area in a decentralized way. A clear differential factor of the existing DATMO algorithms is the typology of sensors used, such as cameras, LiDAR or radar sensors and combinations of those [5,6,7]. Although vision systems can provide dense color data and very rich features, they are very sensitive to reflections, light changes, and harsh climatology conditions such as heavy rain or fog. Radars can directly provide motion information, but they can become very noisy and these sensors are not commonly integrated in existing AGVs. On the contrary, laser sensors are more robust to illumination changes and adverse weather conditions, providing stable measurements. From them, 3D LiDARs are obtaining more presence in autonomous vehicles [8,9,10,11]. However, they are expensive sensors and they provide much information that mobile platforms can struggle to process in real time, apart from the fact that they are not usually available in currently existing AGV platforms. In contrast, 2D-LRFs are much cheaper and can already be found placed in current AGVs for extra security purposes such as emergency brake activation.

**Figure 2:**
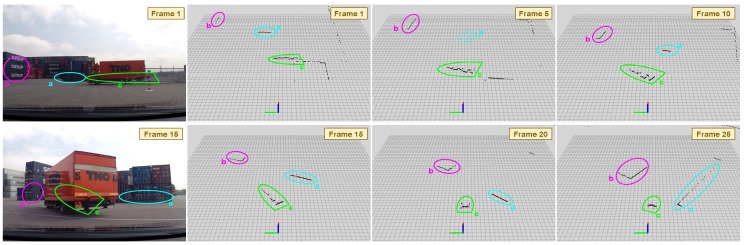
2D-LRFs mounted on mobile platforms provide scarce measurements that are sometimes hard to interpret even for the human eye. We show several frames of a SICK LRF in a port environment performing the vehicle following task. It can be appreciated how detected objects *a* and *b* vary drastically in size and shape due to occlusions and perspective changes. In the same manner the moving truck *c* has a completely different shape comparing frame 1 to frame 15. Best viewed in color.

Using only 2D-LRF information is a challenging problem due to the little and scarce data perceived from the environment. For example, a small object occluding a bigger one can force miss-detection of the second object as two different ones in the scene. However, further challenges arise when using these sensors from a mobile platform, as can be observed in Figure 2. Occlusions and abrupt changes of perspective will be generated, deriving in more complex scenarios from which detected objects could easily appear, even to the human eye, as dynamic ones.

In this paper, we propose a low-cost and robust DATMO algorithm that can exploit the little information provided by existing in-vehicle 2D-LRFs obtaining more than 98% of total system recall while working in real time. This article extends the work presented in [12]. Apart from refactoring our code to increase the full system speed, other elemental changes have been made. More specifically:

- We have developed and simulated a virtual port environment to account for the lack or real existing data. With it, we can perform further experiments that validate our system generalization. We have run a full set of common autonomous transportation situations with several vehicles such as approaching intersections, joining/leaving platoons, overtaking maneuvers, etc.
- We can run our system employing different sensor configurations on the platform, demonstrating that our system can easily be introduced in different existing AGV models and therefore its hardware generalization capabilities.
- We have increased the detector accuracy. A dynamic threshold according to the detected distance is introduced on the matching step. Moreover, due to the new complete sets of simulations performed, we are now capable of detecting more robustly different moving objects in ports, such as straddle carriers, loaded/empty AGVs, automated trucks, cars, etc. We have also improved our method for propagating the reference point of detected objects through time, gaining robustness against changes of perspective and occlusions. These changes will be detailed in Section 3.
- We have boosted the general performance of our Multi-Hypothesis Tracker (MHT). Our local coordinates tracking has been improved obtaining better observations and velocity estimations from the AGV point of view, which helps in further filtering static objects as well as eliminating false positive detections. Being able to observe much more different situations in the new virtual environment, we obtain better insights to adjust our parameters. We are now able to hold tracks for a longer time when objects get occluded or are temporally not detected. We have also improved our target grouping strategy by merging the previously generated targets according to track similarity both in terms of velocity and distance. These changes will be detailed in Section 3.2.
- We perform a comparison of the new DATMO system over the real dataset with respect to [12], analyzing the contributions of the different improvements performed.

The rest of the article is organized as follows. Section 2 reviews the state of the art of DATMO systems focusing on those works using only single-layer rangefinder sensors. Next, Section 3 details the different components of the presented DATMO system, especially stressing the new additions. In Section 4 we present the developed virtual environment along with a representative set of simulated scenarios. New results from our real data collected on the port of Hengelo in the Netherlands are also presented and analyzed. Finally, conclusions are presented in Section 5.

## 2. State of the Art

DATMO has been a problem studied for long time [7,8,13,14,15]. As a general rule, existing approaches divide the process in two separated tasks: firstly, a detection algorithm finds objects in static frames at fixed time; secondly, those objects are tracked in time by using for example, variants of the Kalman Filter [16] and MHT [17,18].

### 2D Laser-Based Detection

Detection methods from 2D-LRF sensors usually use segmentation or clustering algorithms to divide the range measurements into meaningful pieces from which different features or cues are extracted. In this way, [19] focus on extracting lines and other features from the scans taking advantage of the ordered set of measurements provided by LRFs. A more deeply comparison on line extraction algorithms is presented by Nguyen et al. in [20]. Conversely, [21] propose methods for scan segmentation and matching in polar coordinates. Of special interest about segmenting laser scans, is the work of Premebida et al. [22]. It describes different algorithms for segmenting 2D-LRF information and presents methods for feature detection and geometric primitive extraction, such as lines, circles, and ellipses. Complementary geometric primitives are extracted by Mertz et al. in [14]. They present a DATMO algorithm which use detected corners as features that are less susceptible to changes of viewpoint. Aiming to obtain more abstract and descriptive features in 2D lasers, [23] proposes a set of new features encapsulated in what they called the FLIRT descriptor. These features claim to have a similar repeatability and matching performance than interest points of much richer domains such as cameras (SIFT or SURF features). However, they are computationally expensive, and therefore do not match our requirements of easy computation for real-time working in simple hardware.

Once the information of the sensor has been processed it follows the object detection step, which can be roughly summarized in two main approaches, to be, model-based and model-free detectors.

Model-based approaches aim to recognize objects with a known model, basically containing several of the extracted features or a defined shape. These methods have (or learn) prior information about the classes to be detected and search for them at every frame. In this way, Arras et al. [24,25] focused on indoor people detection and detect legs of people on 2D-LRFs by extracting 14 different features from the range measurements and learning a boosted classifier model of people legs applying an adaboost strategy. Due to its prior knowledge of the classes, model-based detection can solve some of the inherent complications existing when dealing with the scarce data provided by 2D-LRFs. Several clusters produced due to over-segmentation or occlusions can be grouped into one if they fit in the same object model, as it could be done with object *a* on Figure 2. Moreover, it can alleviate perspective changes, so that helping to deal with cases such as the object *c* on Figure 2. In this line of work, [26] models the geometric properties of the tracked vehicles and impose shape estimations on them, obtaining more stable reference points of the vehicles through the time. However, unknown and potentially hazard objects may not be included in the set of pre-defined models and therefore not detected when using these model-based approaches.

On the other hand, model-free detection approaches such as [14,15] do not have any prior knowledge (or model) about the classes that can be found in the scene. Therefore, there is no restriction on the kind of elements it can detect, which means that objects can be found regardless whether it is a vehicle, a person, or any other unknown entity. Due to this fact, we have implemented our detector aligned to these model-free approaches. The main drawback, however, is that these kinds of approaches rely deeply on the correctness of the segmented scans and the posterior extraction of high-quality features, therefore we pay special attention to this point.

As already commented, two-step DATMO paradigms commonly focus on detecting objects in static frames, leaving for the later tracking phase the task of associating them through time. Yet, tracking all the extracted features and segmented objects becomes computationally very expensive, which results in slow performance in a mobile platform. In our DATMO system we aim to alleviate this processing load by performing a fast-matching step of elements between frames in the detection stage, therefore filtering out objects that are strongly considered as static.

#### Tracking by Detection

The main objective of a tracker algorithm is to assign a consistent label detected objects through time, while retrieving other useful information such as orientation, velocity, size, or shape. It is commonly done in three steps: target state estimation, object association and target correction. The first stage, propagates and predicts the new positions of the existing tracks according to their state vector and the ego-motion. In the second phase, current detections are associated with propagated tracks. Finally, a target correction step updates the matched targets according to the association data.

Literature tracking strategies commonly fuse sensors (e.g., cameras, LiDAR). In this way, ref. [27] tracks multiple objects by fusing information from several overlapped cameras and a low-resolution LiDAR and creates a learning policy from Markov Decision Processes (MDPs) to obtain the appearance, life, and disappearance of the targets. On the contrary, we seek to use the minimum possible data to build a low-cost yet robust algorithm that can work independently in case of other sensor failures. A comprehensive guide with several proposed approaches for object tracking is presented in [28]. We will focus here on reviewing statistical single and multiple point tracking algorithms, which take into account uncertainties in the measurement of the object state gaining robustness against the noises from sensor measurements and motion prediction, as these are the most related to our work.

A general approach for the target estimation step is to consider that the noise and the own object state have a Gaussian distribution, so that the optimal state can be calculated taking advantage of the recursive structure of Kalman Filters (KF). Although KF for tracking points have been vastly used since long time ago, they may provide weak estimations when the object state is not following a Gaussian distribution or in the presence of motion models with strong non-linearities. These limitations can be overcome by using Particle Filters [29], although increasing the computational burden.

If multiple objects are meant to be tracked, a one-to-one association between a certain set of detected objects and the previous known state vectors must be done. In the literature different methods have been applied for this, from the simple Nearest Neighbor to more complex ones such as MHT [30] or Joint Probabilistic Data Association Filtering (JPDAF). Extensive reviews of several statistical data association and fusion techniques can be found in [31,32]. In our approach, we have chosen to use MHT based on Kalman Filters (MH-KF), as it provides us with a good trade-off between accuracy and low computation requirements.

Contemporary to our work, [33] proposes to overcome the appearance change problem that exists when using only 3D LiDAR information and presents a tracking algorithm based on L-shaped detections. However, we are focused on obtaining a robust and lighter tracking strategy, only using 2D-LRFs without the need for any model. In this way we do not only track certain shapes, but we are able to dynamically follow visible corners, lines or even the target’s centroid if none of the previous appear. Moreover, we overcome the benefit that model-based approaches have when dealing with partial occlusions and perspective changes by employing novel track grouping techniques which help us on updating the size and other features of the target. We also include policies to have a better control of the confirmation, hold and deletion of the tracked moving targets, allowing us to reduce the number of false positives and false negatives. The new set of virtual simulations performed in this version with respect to [12], allows us to better adjust these control parameters improving the real results.

## 3. DATMO in Port Environments

The exceptional cargo handling activity of port environments motivates us to design a specialized DATMO algorithm for this environment. Port and cargo terminals are known to be traditional scenarios in which changes on the work chain are introduced very gradually, mainly due to the elevated investment needed. Our approach tries to build up a fully working, robust and low-cost system with minimum intervention over existing AGVs. We therefore reduce the number of sensor dependencies to the minimum and use already existing single-layer LRFs and odometry sensors as only input.

**Figure 3:**
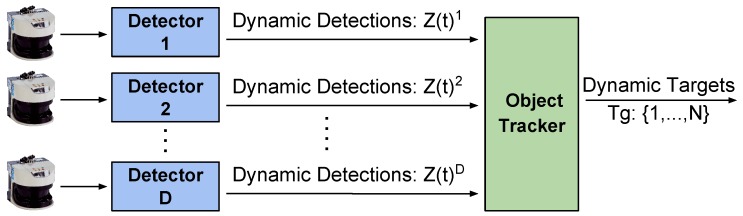
General DATMO proposed schema. Our system can now deal with several LRF sensors placed in the AGV. For each one, a moving object detector is instantiated having as only inputs the range measurements and the vehicle’s odometry. The MH-Tracker receives a list of dynamic objects from each of the detectors and tracks them through time, retrieving their speed, direction and status. Section 3.1 and Section 3.2 detail respectively the internal processes performed by our detector and tracker modules.

Our DATMO approach uses the common two-step paradigm: firstly, we perform detections in a model-free style; secondly, we employ an MH-KF tracker to follow the dynamic obstacles along the time. The core of our algorithm is similar to the one presented in [12], in which we have introduced some novelties. We have refactorized the full code so now communication between modules is more effective and the speed of the general system is increased. Moreover, we can now easily integrate information from several 2D-LRF sensors in the system, allowing different placement configurations as well as not limiting the number of sensors. A general schema of the system working with “*D*” 2D-lidars is presented in Figure 3. As can be seen, for each sensor, the system launches a detector instance which filters the detected objects passing to the final tracking the ones considered as dynamic. The MH-Tracker receives the dynamic objects information from each detector and fuse it, being now able to easily group elements from different detectors.

Specific changes have been done in our detection step, by introducing a dynamic threshold that allows better segmentation of further objects in which LiDAR measurements are sparser. In addition, the tracking phase has also been optimized and specific changes introduced. For example, we now perform our grouping strategy over existing tracks attending to similarities both in the velocity and spatial situation of currently generated targets. If a group already exists, we can now directly associate several new detections to it, recovering in this way from small occlusions. Finally, we allow generalized the use of our DATMO system with different sensor configurations, as will be shown in Section 4.1.

In this Section we will expose in more detail the different blocks that take part in our improved DATMO system, remarking specific changes with respect to [12].

### 3.1. Detecting Moving Objects with Single-Layer Laser Scanners

**Figure 4:**
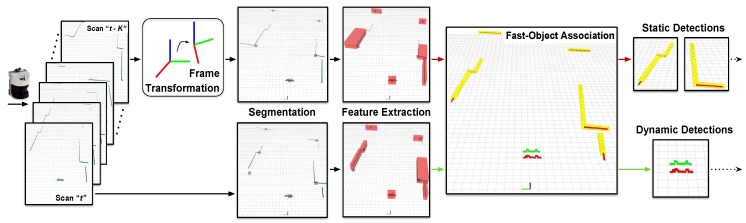
General schema for detecting moving objects with single-layer laser rangefinders (LRFs). We segment the laser measurements using a dynamic threshold and extract different geometric features from each cluster obtained. A fast-matching step is later performed between time-separated scans to filter out static objects, leaving to the next tracking step only the elements of the scene considered as dynamic. Thus, we can reduce between two and five times the objects arriving to the tracker.

We perform a model-free detection step to detect any object regardless its nature or shape, which is essential in the constantly changing and dynamic environment of port terminals. Our detection approach contains several processes, as depicted in Figure 4, that are detailed in this section.

#### 3.1.1. Input Data Pre-Processing

Information provided by LRF sensors can contain multiple echoes (e.g., when a laser ray pass through a window), or to measure the reflectance intensity (which give information about the obstacle’s material). For the shake of generalization and allowing compatibility with different sensors, we only use laser-range information and, in the presence of several echoes, we only account for the first range measurement. Let $Sc_{t}$ be the range information received by an LRF at time *t*. It can be represented as a set of points in polar coordinates as $Sc_{t}=\left\{r_{i},\alpha_{i}\right\},\;i\in \left[1,S\right]$, where *r* is the range distance measured, α is the bearing angle, and S is the number of laser measurements in our filtered scan. In this pre-processing step we also eliminate outliers according to the sensor specifications. In addition, we filter the range laser measurements discarding the ones that are further than 50 m, which we will consider as the maximum interaction distance of the vehicle.

Our object detector can detect moving objects by comparing and matching frames separated by a time window. We therefore store the scans on a buffer of *k* frames, which size will depend on the scenario where the system is deployed as well as the LiDAR sensor frequency. To choose a correct buffer size is of capital importance, as other parameters used in the detection step will directly depend on this one in order to optimize our fast-matching capacities.

As our own vehicle is moving, we need to estimate our motion to compare objects from scan $Sc_{t}$ with previous ones from $Sc_{t-k}$ from the same reference point. Odometry sensors, although they are not very precise when calculating long-term trajectories, provide sufficient information of the ego-motion of the AGVs in between scans. We first transform both scans to a common reference frame on the vehicle ($\cdot^{veh}$), where the odometry data is also referred to, obtaining $Sc_{t}^{veh},Sc_{t-k}^{veh}$. Next, we apply to the older scan the transformation given by the motion measured by the vehicle, getting the scans in a comparable reference frame $Sc_{t}^{veh}$ and $Sc_{t-k}^{veh+o}$. For the shake of simplicity, from now on we will omit the superscript index, as in further steps all the scans will be under the same vehicle’s reference.

Both our real and simulated cargo terminal contain vehicles moving at maximum speeds of 6 m/s carrying LRF sensors that provide data at 10 Hz. With this scenario we have chosen to set the size of our buffer to k=10, thus matching elements from the actual scan $Sc_{t}$ and the ones obtained one second (10 frames) before in scan $Sc_{t-10}$, which represents a maximum displacement of 6 meters.

#### 3.1.2. Scans Segmentation

Before performing the object matching, each scan is segmented in separated elements by spatially grouping sets of points. We speed up this process by taking advantage of the sequential order of range measurements provided by LRF sensors using the Point-Distance-Based Segmentation (PDBS) [22]. As the angular step of common LRF sensors is very small, the Euclidean distance between two consecutive scan points can be easily approximated by just calculating the $\ell_{1}$ range distance $\ell_{1}\left(r_{i},r_{i-1}\right)=\left|r_{i}-r_{i-1}\right|$, which is much faster. Therefore, points in the scan are grouped in different objects if the $\ell_{1}$ distance between two consecutive ranges is higher than a given threshold $Th_{seg_{r}}$. In [12] we used a fixed threshold $Th_{seg}$ for this task, but analyzing the new simulations performed here we observed that a fixed threshold tends to over-segment objects located far away. Thus, we have improved this step and set this threshold value as a function of the range such as $Th_{seg_{r}}=Th_{seg}\left(r_{i}/100+1\right)$. In our final experiments, initial threshold $Th_{seg}$ is set to 0.3 m and therefore it grows 1.5 times up to 0.45 m at the maximum interaction distance of 50 m.

**Figure 5:**
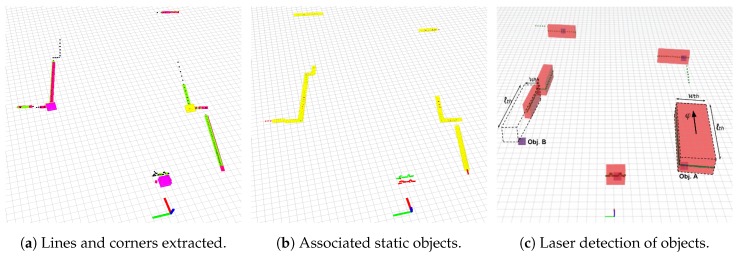
Objects detected and extracted geometric primitives. (**a**) shows segmented elements from two overlapped scans $Sc_{t}$ and $Sc_{t-k}$ and their respective lines and corners extracted. Green lines and yellow corners belong to the actual scan $Sc_{t}$, whereas purple lines and red corners are extracted from the past $Sc_{t-k}$ scan. We can clearly appreciate how corners and lines are representative features, being corners more reliable. (**b**) shows in yellow the segmented objects matched, so that considered as static. A dynamic detected object in the middle is shown without a match, and both previous and current scans can be appreciated in red and green, respectively. (**c**) represents the features extracted from detected objects, where we can observe the reference propagation of *Obj. B*. Best viewed in color.

At this initial point, every obtained cluster of LiDAR points will correspond to a segmented object without any specific model. To represent them, each object is initially parameterized by the following state vector:

$$obj_{j}={\left[ref_{act},wth,lth,\varphi ,np\right]}^{T}\;,$$

where j∈[1,J] refers to each segmented object, $ref_{act}$ is the actual reference frame of the object (in Euclidean coordinates with respect to our vehicle’s frame) as will be detailed in Section 3.1.4, wth and lth are respectively the object’s width and length, φ is the orientation with respect to the longitudinal axis, and np is the number of laser points that the object contains. Figure 5c shows an example of this parametrization over a real scan.

#### 3.1.3. Geometric Primitive Extraction

When perceiving only a single slice of range data from the environment, one of the biggest problems that we need to face is the absence of trustworthy reference points in partially observed or occluded objects. The two most-upper red blocks of Figure 5c, which are partially visible without any additional shape cue, clearly exemplify this situation. However, real port environment objects such as containers, buildings or vehicles have a strong geometrical structure containing well defined straight lines and corners. To get reliable features, we extract for each segmented object in $Sc_{t}$ and $Sc_{t-k}$ its inherent geometric primitives [14], such as lines and corners, as can be seen in Figure 5a.

In contrast to [12], we have experimented in this article with two different line extraction approaches as in [20]. On one side, we perform a linear regression over small subgroups of consecutive points. We next develop a line-merging procedure based on angle similarity between adjacent lines extracted, building bigger lines. For these longer lines obtained, we finally assure that the error from a new regression performed over all the corresponding points will remain under a certain threshold. On the other hand, we have also used the Hough Transform to find the best fitting lines in the full set of points. Although accuracy of the Hough Transform is slightly better, this method requires more computational power that affects our global processing speed. For this reason, in our final algorithm we keep using the linear regression method which better fulfills the speed vs accuracy trade-off.

We parametrize the structural lines of every element in a scan as follows:

$$obj_{j}\left(line_{l}\right)=\left[pt_{init}\left(x,y\right),\;pt_{end}\left(x,y\right),\;\epsilon ,\;\vec{\theta }\right]\;,$$

where l∈{1,…,L} represents each of the lines of an object, $pt_{init}$ and $pt_{end}$ are respectively initial and final Euclidean points, ϵ is the obtained linear regression error and $\vec{\theta }$ is the line’s director vector.

Apart from structural lines, we extract corners of each of the detected objects given that they exist. In this way, corners are created when two lines of the same object intersect with an angle larger than π/6. We define this geometric primitive in a state vector as

$$obj_{j}\left(corner_{c}\right)=\left[pt_{corner}\left(x,y\right),\;\rho ,\;\gamma \right]\;,$$

being c∈1,…,C each corner of the object, $pt_{corner}$ its corresponding Euclidean position, ρ its orientation and γ its aperture. Notice than in contrast to [12], we now group our lines and corners inside the containing object and not the other way around, which significantly helps on performing faster object matching between scans, speeding up the full system.

Apart from these geometrical features, other useful attributes such as perimeter (P) and area (A) of each object are extracted. In addition to [12], we also extract the polyline that describes the object’s bounding box, which keeps track of the fully observed shape of the objects, as can be appreciated in Figure 5c. For defining this polyline, the principal component of the cluster of points is obtained. This can be done by using Principal Component Analysis (PCA), although it requires additional computations which would delay our execution, so we simplify this process by selecting the longest line of the object. Next, we rotate the object clockwise to a zero position according to its principal component and select the points with maximum and minimum Euclidean positions, which will define our polyline. Finally, each object is therefore defined by combining its spatial features and geometric primitives as:

$$obj_{n}=\left[ref_{act},\;wth,\;lth,\;\varphi ,\;np,\;L,\;C,\;P,\;A,\;polyline\right]\;.$$

#### 3.1.4. Fast Object Matching and Reference Propagation

The final step of our detector exemplifies our efforts to reduce the computational burden of the next tracking stage. Objects from scans $Sc_{t}$ and $Sc_{t-k}$ (both in the vehicle’s reference frame) are matched to perform a fast filtering of static objects, therefore leaving for the tracker the detected elements with better chances to be dynamic. Each structural feature (including lines and corners) from scan $Sc_{t}$ is compared with the ones of scan $Sc_{t-k}$ to match them. If a matching is obtained between an object in the actual scan with another in the older, it means that the object has not moved so it is tagged as static. This simple approach allows us to reduce between 2 and 5 times the number of objects analyzed by the tracker, as it is shown in Section 4.

We use a logical programming approach for performing the object associations, attending to the presence and the strength of each of the geometrical features extracted on the objects. As corners are hardly invariant features, if two objects under comparison produce a corner match, we associate them on both scans and directly tag them as static. Formally, a corner “*A*” from an object of the scan $Sc_{t}$ and a corner “*B*” from one of the scan $Sc_{t-k}$ are initially matched if the following logic rule is accomplished:

$$\ell_{2}\left(pt_{corner}^{A},pt_{corner}^{B}\right)<thr_{dist}\;\&\&\;(\;\ell_{1}\left(\rho^{A},\rho^{B}\right)<thr_{\rho }\;\;\|\;\;\ell_{1}\left(\gamma^{A},\gamma^{B}\right)<thr_{\gamma }\;)\;,$$

which states that the $\ell_{2}$-Norm between the corners’ reference point must be less than a defined distance threshold, and that at least one of the $\ell_{1}$-Norms from the aperture or orientation corner features needs to be within $thr_{\gamma }$ and $thr_{\varphi }$ ranges, respectively.

In the absence of corners, two objects can be matched by its structural lines, although lines may suffer from more variability due to changes on perspective and occlusions as appreciated in Figure 2. Formally, objects are associated by means of it lines only if the angle between their orientations is within $\hat{thr}$ range and at the same time the $\ell_{2}$-Norm between their initial or final points is below the given distance threshold $thr_{dist}$, as states:

$$\hat{{\vec{\theta }}_{line}^{A},{\vec{\theta }}_{line}^{B}}<thr_{\theta }\;\&\&\;(\;\ell_{2}\left(pt_{init}^{A},pt_{init}^{B}\right)<thr_{dist}\;\;\|\;\;\ell_{2}\left(pt_{end}^{A},pt_{end}^{B}\right)<thr_{dist}\;),$$

Figure 5b shows a real example of objects matched from two different scans taken in a real port. The current scan is shown in green, the past one in red and the elements tagged as static appear in yellow color. As can be appreciated, a dynamic obstacle in the center is not matched, and points from both previous and current scans are visible in red and green, respectively.

One of the biggest challenges that our model-free detector inherits from the little information provided by single-layer LRFs is that the same object seen at two particular times can be represented with a different reference point due to the changes of perspective. Along with the fast-matching step, we also propagate strong object’s reference points from partially seen obstacles, as well as to update information about the perimeter, area, and the polyline of the objects.

Object matched by means of corners are considered very reliable so that the corner position is kept as the actual reference point ($ref_{act}$) for future frames. Given the case that an object is matched from two scans in which only a line of the object is observed in one scan and a corner in the other (e.g., due to a perspective change), the reference point of the object is updated to the corner’s one. On the contrary case, if an existing object with a corner is matched with an object in a newer scan having only a line, the initial corner is propagated and kept as the reference point on the object’s state vector.

**Figure 6:**
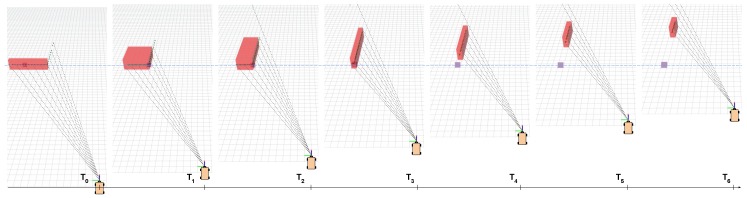
Size update and reference propagation of an object over associated detections with partial visibility. In $T_{0}$, we observe the object with no reliable features, so that its centroid is tagged as reference point. From $T_{1}$ to $T_{3}$, the object is visualized from a different perspective, and the reference is updated to the visible corner. In times $T_{4}$, $T_{5}$, $T_{6}$, the object is partially observed but, as it is matched during the fast-matching step, the old corner is propagated, and the reference point updated. Best viewed in color.

Figure 6 exemplifies a real situation when the corner’s reference of an observed object is propagated. Lacking any better reference, initially the object is tracked in $T_{0}$ from its centroid. Once a corner is discovered, our system updates the track and starts referencing the object from its corner position, as seen from $T_{1}$ to $T_{3}$. When the corner is not visible anymore, but the object is still matched between scans, we propagate the previous corner’s position so that it will reference the now partially visible static object, as seen from $T_{4}$ to $T_{6}$.

### 3.2. Tracking Dynamic Objects in Port Environments

**Figure 7:**
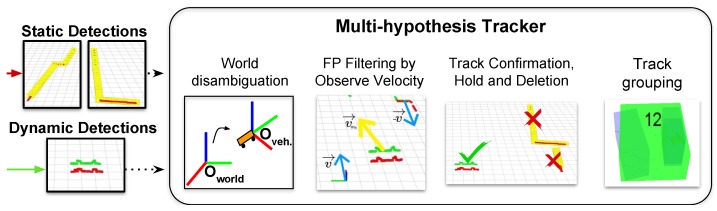
Modules included in our MH-Tracker system. Our tracker works in local coordinates from the ego vehicle, so an initial coordinate transformation is performed to the dynamic detections. In this local frame, we filter false positively detected dynamic objects attending to their observed velocity. We further introduce our track confirmation, hold and deletion functions as well as our track grouping, which is based both on object spatial location and target velocities. Best viewed in color.

The second stage of our DATMO system receives the detected dynamic obstacles and track them through the time. We employ the core MHT of [12], which combines Reid’s algorithm [30] along with the prediction used in [34] that takes into account a window of the previous tracks positions to calculate the average track velocity. We also integrate in our MHT the ability to run from the moving reference frame in local coordinates as in [35], which is essential for our application. In addition to [12], in this article we have improved the confirmation, hold and deletion policies for moving targets as well as the grouping techniques. In this way, we are now able to better deal with partial occlusions and perspective changes. A general schema of the full proposed tracker is shown in Figure 7. In the next sections we detail the our MHT formulation and analyze the different modules along with the new introduced features and functions.

#### 3.2.1. Multiple-Hypothesis Tracking

Our MHT have a set of multi-dimensional observations at each time *t*, represented by $Z\left(t\right)\equiv \{Z_{m}\left(t\right),m=1,2,\ldots ,M_{t}\}$, and a set of target states represented as $x\left(t\right)\equiv \{x_{n}\left(t\right),n=1,2,\ldots ,N\}$. For the shake of fast computation and given the little information provided by LRF sensors, in our case the observations consist on the object’s reference points and their associated covariances. Targets are composed by positions, velocities ($v_{x}$, $v_{y}$), and their respective covariances. Due to its recursive structure and lower computational requirements we use a Kalman Filter for the propagation and correction steps of the tracked targets (x(t)). The possible choice of a particle filter along with the MHT tracking increase the computational cost affecting the final real-time performance.

In the tracker association step, a distance between the detections Z(t) and the propagated targets x(t) needs to be measured. We have here discarded the use of Euclidean distance in favor of Mahalanobis distance which, although is computationally more expensive, it is a more powerful measurement leading to much better decision boundaries. In this way, we associate a detection and a target if their Mahalanobis distance is below a threshold as stated in:

(1) $$\begin{array}{c} {\left(Z_{m}-H\bar{x}\right)}^{T}{\left(H\bar{P}H^{T}+R\right)}^{-1}\left(Z_{m}-H\bar{x}\right)\leq \eta^{2}\;; \end{array}$$

(2) $$\begin{array}{c} \begin{array}{c} Z_{m}={\left[x,\;y\right]}^{T}\;;\; \end{array} \end{array}\begin{array}{c} \begin{array}{c} \bar{x}={\left[x,\;y,\;v_{x},\;v_{y}\right]}^{T}\;; \end{array} \end{array}$$

(3) $$\begin{array}{cc} R & =\left[\begin{array}{cc} G_{x^{2}} & G_{xy}\\ G_{yx} & G_{y^{2}} \end{array}\right]\;;\; \end{array}\begin{array}{cc} \bar{P} & =\left[\begin{array}{cccc} G_{x^{2}} & G_{xy} & G_{xv_{x}} & G_{xv_{y}}\\ G_{yx} & G_{y^{2}} & G_{yv_{x}} & G_{yv_{y}}\\ G_{v_{x}x} & G_{v_{x}y} & G_{{v_{x}}^{2}} & G_{v_{x}v_{y}}\\ G_{v_{y}x} & G_{v_{y}y} & G_{v_{y}v_{x}} & G_{{v_{y}}^{2}} \end{array}\right]\;;\; \end{array}\begin{array}{cc} H & =\left[\begin{array}{cccc} 1 & 0 & 0 & 0\\ 0 & 1 & 0 & 0 \end{array}\right]\;; \end{array}$$

where $Z_{m}$ is the position of the current detection, $\bar{x}$ is the propagation of the target state, H is the measurement matrix, $\bar{P}$ is the covariance matrix of the propagated target, R is the covariance matrix of the detection and $\eta^{2}$ is the association threshold.

In case that a detection could be associated with different targets, our MHT tracker calculates all the possible association hypotheses along with its own probability following the core equation of [30]. For a better comprehension we present the full formula as:

(4) Pit=η″PdetNdet(1−Pdet)NTGT−NdetβfalNfalβnewNnew  Ndetector(Zm−Hx¯,B)Pit−1Pnc(Nnc)Ph(Nna)

(5) $$B=H\bar{P}H^{T}+R\;,$$

where ${P_{i}}^{t}$ is the probability of the actual hypothesis; $\eta^{\prime \prime }$ is a normalization term which serves to make the sum of all the probabilities of the current hypotheses equal to 1; $\beta_{fal}$ and $\beta_{new}$ are the Poisson probability distributions for the cases of false alarms and new targets; $N_{fal}$ and $N_{new}$ are respectively the number of detections associated with false alarms and the number of detections associated with new targets; $P_{det}$ and $1-P_{det}$, are the probability of detection and the probability of not detection; $N_{det}$ is the number of detections associated with existing targets and $N_{TGT}$ is the number of the existing targets; $N_{detector}\left(Z_{m}-H\bar{x},B\right)$ is the Gaussian probability distribution of the detections for the detector; and ${P_{i}}^{t-1}$ is the probability of the previous hypothesis from which derives the current hypothesis. All these terms share the values stated at [30], where the reader is referred for a deeply explanation.

When there exists any association conflict within a new target hypothesis, [30] takes the hypothesis with higher probability and propagates it through time in a tree. Conversely, we simplify and speed up this process in this paper by taking only the hypothesis with the highest probability therefore discarding the others and pruning the tree expansion.

Additionally, for calculating the probability of each hypothesis as in Equation (4) we included in [12] two additional terms: (1) $P_{\mathrm{nc}}\left(N_{nc}\right)$, which is the probability distribution for the not confirmed targets (added to confirm the targets as dynamic objects); (2) $P_{h}\left(N_{na}\right)$ which is the probability distribution to hold dynamic targets. In this work, after further experimentation in our new simulated environment, we have redefined our confirmation function as an exponential one, differently than the one used in [12]. Nevertheless, both functions are detailed in Section 3.2.3 for the shake of full system comprehension.

#### 3.2.2. Filtering Static Objects by Velocity

Although our detector step for fast-matching objects between scans reduces the number of false positively detected moving obstacles between 2 and 5 times, some of them will still exist. In this way, our tracker algorithm also filters false positive detections (static objects) attending to their observed velocity. Our DATMO system works in local coordinates on the reference frame of the ego vehicle. Therefore, an object standing static in the environment will be observed as having our same velocity but contrary direction in this local frame. In the same manner than in [12], we filter objects with the following local velocity with a ±0.5 m/s margin:

$${{\vec{V}}_{obj}}^{local}=-\left({{\vec{V}}_{veh}}^{world}+{{\vec{W}}_{veh}}^{world}\times \vec{d}\right)$$

where ${{\vec{V}}_{veh}}^{world}$ and ${{\vec{W}}_{veh}}^{world}$ are respectively the linear velocity and the angular velocity of the vehicle in the world coordinates, and $\vec{d}$ is the distance between the vehicle and the tracked object. The super index local and world means the local coordinates of the vehicle and the global coordinates of the world.

#### 3.2.3. Confirmation, Hold and Deletion of Moving Object Tracks

The confirmation and deletion terms $P_{\mathrm{nc}}\left(N_{nc}\right)$ and $P_{h}\left(N_{na}\right)$ of Equation (4) allow us to have a better control of the confirmation, hold and deletion of the moving targets.

The term $P_{\mathrm{nc}}\left(N_{nc}\right)$ is introduced to deal with false positive detections (not real moving objects) by imposing the target confirmation with a slower rate than other tracker approaches. Arras et al [25] solve this by having two types of targets, ones related to confirmed objects (approved targets) and the others related to not confirmed objects or no-objects (free targets). However, we define this term by implementing two separated states of the same target, thus avoiding having twice the targets and related probabilities. In [12] we formulated this confirmation function in a linear manner. After carefully analyzing our new simulated scenarios, in this article we model this term with an exponential probability distribution. In this way, we set the confirmation probability to grow exponentially as long as the track has a detection associated with it according to:

(6) $$P_{\mathrm{nc}}\left(N_{nc}\right)=\left\{\begin{array}{ccc} 1-e^{-\lambda \cdot N_{nc}} & if & N_{nc}\geq 1\;\mathrm{and}\;\mathrm{target}\;\mathrm{not}\;\mathrm{confirmed}\\ 1 & if & \;\mathrm{target}\;\mathrm{confirmed} \end{array}\right.$$

where $N_{nc}$ is the number of times that the target has a detection associated with it and λ controls the growing speed of the probability, balancing how fast a target is confirmed. In our case, we use λ=0.02.

Equation (6) only applies when the target is still not confirmed and increase its confirmation probability each time that a detection gets associated until it surpasses a confirmation threshold, so then it is confirmed. We have set this confirmation threshold to 0.9 for our experiments in the port environment. With this, we gain protection against false positive detections by moderating the confirmation, contrary to other binary approaches that confirm targets with just one association.

**Figure 8:**
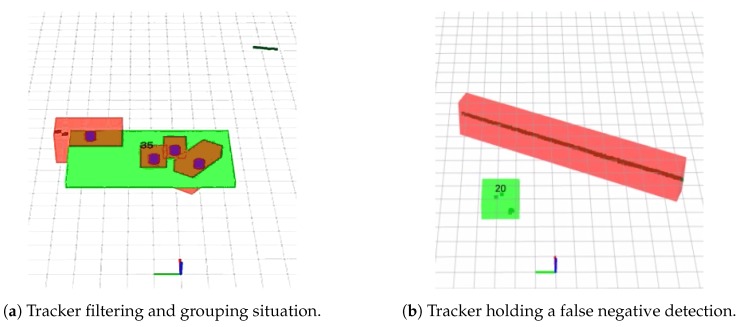
Tracking grouping and holding cases over real data. In green the final moving target with its identifier. Red objects are segmented elements and blue cylinders are dynamic elements tagged by the detector. (**a**) shows how several over-segmented objects are grouped into one unique track in the center of the image. In addition, our tracker can filter a false positively detected dynamic object as seen in the top left corner. (**b**) shows our tracker holding a moving vehicle in a false negative situation.

To deal with false negative detections (no detections of moving objects) our tracker holds the existing moving targets during short periods of time. This is done as in [12] by slowly decreasing the target probability with the introduction of a probability distribution for holding dynamic targets:

(7) $$P_{h}\left(N_{na}\right)=\left\{\begin{array}{ccc} P_{h}^{N_{na}} & if & \;\mathrm{target}\;\mathrm{not}\;\mathrm{associated}\\ 1 & if & \;\mathrm{target}\;\mathrm{associated} \end{array}\right.$$

where $P_{h}$ is the probability of holding a target, set to 0.99 and $N_{na}$ is the number of targets without dynamic detection associated. A target is deleted when its probability decreases under an elimination threshold that we have set to 0.4 for this paper for better generalization purposes after running our new set of simulations. Figure 8b shows a real experiment where the tracker can hold a target when no detection exists (no red boxes exist for the dynamic vehicle ID20). If the detection reappears and is again associated with the target, it will keep its ID number.

#### 3.2.4. Track Grouping

Due to occlusions or errors in the segmentation step of our detector, sections of a bigger object can be detected separately. Moreover, as in this article we include the possibility of using several 2D-LRFs in our DATMO system, the same object can be detected from different sensors and each one with a different reference point. We can merge this multiple or partially detected objects in a new track ID attending to both the actual position of each element as well as its tracked velocity. In contrast to [12], we included here the grouping by velocity option so that making our algorithm more robust. We use the following equations to group the tracks using similarities in distance and velocity:

(8) $${\left(H{\bar{x}}_{1}-H{\bar{x}}_{2}\right)}^{T}{\left(H\bar{P_{1}}H^{T}+H\bar{P_{2}}H^{T}\right)}^{-1}\left(H{\bar{x}}_{1}-H{\bar{x}}_{2}\right)\leq {\eta_{d}}^{2}$$

(9) $${\left(H_{v}{\bar{x}}_{1}-H_{v}{\bar{x}}_{2}\right)}^{T}{\left(H_{v}\bar{P_{1}}{H_{v}}^{T}+H_{v}\bar{P_{2}}{H_{v}}^{T}\right)}^{-1}\left(H_{v}{\bar{x}}_{1}-H_{v}{\bar{x}}_{2}\right)\leq {\eta_{v}}^{2}$$

(10) $$H_{v}=\left[\begin{array}{cccc} 0 & 0 & 1 & 0\\ 0 & 0 & 0 & 1 \end{array}\right]$$

notice that Equation (9) group the tracks using velocities. We have separated it from Equation (8), which was used in [12], to further strength the fact that we perform a dual grouping based both on velocities and spatial positions. H shown in Equation (3), is the H measurement matrix defined in the same way as in [30], which allows to group the tracks using its spatial proximity and $H_{v}$ of Equation (10), allows to group tracks according to their velocity. ${\bar{x}}_{1}$ and ${\bar{x}}_{2}$ are the propagated and corrected target states, as shown in Equation (2), and $\bar{P_{1}}$ and $\bar{P_{2}}$ are its corresponding covariances as in Equation (3); ${\eta_{d}}^{2}$ and ${\eta_{v}}^{2}$ are the thresholds that allows the association of different targets of the same moving object, using distance and velocity respectively.

When a new group of targets is generated, the new track takes into account the position and velocities of all its contained targets. We calculate the group velocity, as the average of the target velocities. When a group is generated, we also estimate the size of the final grouped target using two different approaches. On the one hand, when detections come from different sensors and the object areas overlap, we calculate the final shape as an average of each independent detection. On the other hand, when the grouped detections come from the same laser with no overlapping areas, chances are that the object was partially occluded and therefore the independent sizes are summed up. In any case, we propagate the biggest size of each object so that we can keep tracking their real shape while its observed from different points of view.

## 4. Simulations and Experiments

We describe the most meaningful simulations and experiments performed to test and validate our DATMO system. For this article, we have simulated a realistic port environment in which different common situations for autonomous transportation are evaluated. These experiments contain several moving vehicles and different sensor configurations to show the generalization capacities of our system under different conditions. We test as well our system with real data captured in the port of Hengelo, The Netherlands and compare our new results with the ones in [12]. Both scenarios show that our system can keep the track of dynamic objects with just the little information provided by a 2D-LRF under challenging situations such as occlusions and changes of perspective.

### 4.1. Simulated Environment

**Figure 9:**
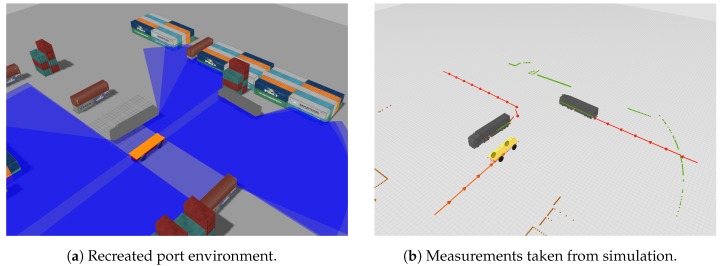
Samples of our simulated environment that recreates real ports conditions. Our simulations include different kind of standard containers, vehicles and other elements such as light posts or road cones. We placed an AGV test platform with Ackermann’s motion model equipped with six SICK LRFs covering $360^{\circ }$. Trajectories for each vehicle in the scene can be separately defined as are shown in (**b**).

We have simulated a realistic environment of a port terminal in which our DATMO system could be validated. The recreated world aims to emulate in a faithful way the harbor conditions, so we have included several vehicles, standard container sizes, as well as poles and additional elements which can be seen in Figure 9. Within this virtual world we have recreated an AGV with Ackermann movement model equipped six LRFs attached to its body which characteristics resemble the SICK LMS511 sensors. The simulated sensors are placed around the vehicle in the following way: four of them are situated on the corners, two pointing forward and the other two pointing backwards; the remaining two sensors are placed on the left and right side of the vehicle. This configuration provides a field of view of $360^{\circ }$, with redundancy on the front and back areas, which are critical for the AGV.

We have thoroughly simulated a set of common autonomous driving/transportation situations that could occur in a cargo terminal, including turns, crossing vehicles, takeovers, occlusions, and vehicle following scenarios. For each situation, we have tested our system performance with different sensor configurations, from having only one frontal LRF providing a field of view of $180^{\circ }$, to employing four LRFs providing $360^{\circ }$. In the next sections we present some of the most relevant simulations performed.

**Figure 10:**
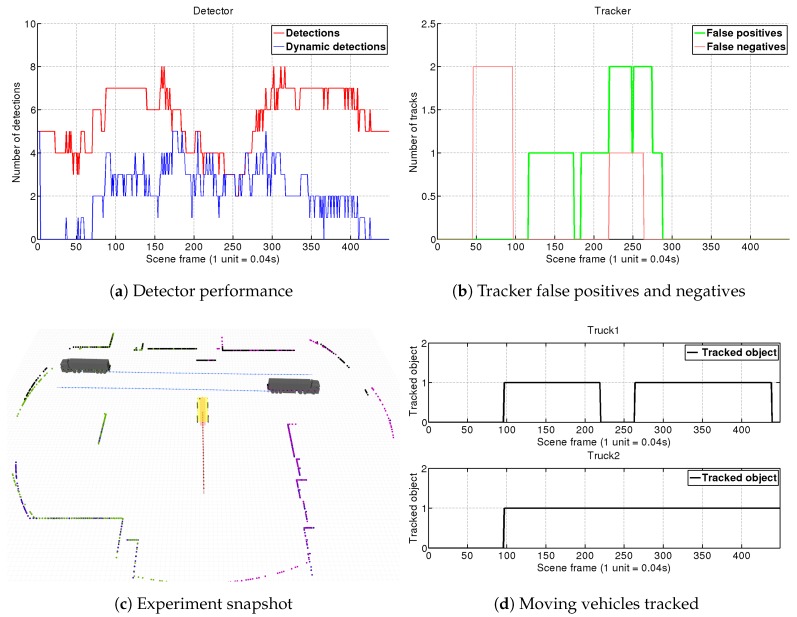
Scene 1. The AGV approximates an intersection where two trucks coming from both sides cross in front of it. After entering in the AGV’s field of view, our DATMO system keeps tracking both vehicles. In the mid part of the simulation, *Truck 2* occludes *Truck 1*, and its target is lost after a second without receiving detections being tracked again when new detections are provided.

For each experiment, we show four different graphics. In the first one (e.g., Figure 10a), we analyze the detector performance by showing the number of total objects segmented at each frame (red line) along with the number of those objects which our detector has tagged as dynamic (blue line). Analyzing this chart through the simulations, we can see how our fast-matching approach for filtering dynamic objects in the detector is consistently reducing the tracker load by two to four times. The second graph (e.g., Figure 10b), shows the tracker false positives (objects tracked as dynamic but actually static), along with the false negatives (objects not tracked as dynamic which actually are). To give an insight of the tracking performance on each sequence, we present complementary charts (e.g., Figure 10d) showing the number of frames along the whole scene in which our DATMO system manage to correctly track each vehicle (true positives per vehicle). Finally, for better understanding each simulated situation, a snapshot reflecting the motions of the different elements in the scene is shown (e.g., Figure 10c), so that conclusions about occlusions or changes of perspective can be extracted.

#### 4.1.1. Scene 1: Two Trucks Crossing

For this simulation we have tested the capacity of our DATMO system with only one single 2D-LRF. In this situation, as seen in Figure 10c, the AGV approaches an intersection where *Truck 1* and *Truck 2* are coming from the right and left sides, respectively. The AGV sees them, stops, and then continues its way. This is a challenging scene with multiple changes of perspective and occlusions.

Results of this simulation are shown in Figure 10. At the beginning both trucks are outside the AGV’s field of view (FOV), and do not enter it until frame 50. The vehicles are detected as moving objects in frame 70 which means a delay of just 20×0.04=0.8 s. It is worth remembering that the detector compares scans separated within 10 frames, so here is comparing frame 70 ($Sc_{t}$) with frame 60 ($Sc_{t-10}$). If no object matching is produced by the detection module, the object is directly tagged as dynamic so intrinsically this step does not suppose any special lag. In this case, the delay is mostly introduced by the segmentation module that needs to discard small groups of far points when the trucks approach our FOV. By frame 95, both objects tagged as dynamic by the detector are confirmed by the tracker. This lag is introduced by Equation (6) which was adjusted for our port environment maximum speeds. Due to the relatively low velocities of the vehicles in the port, we trade-off a slower confirmation of far vehicles for obtaining a system that is more robust against false detections.

This scene contains a full dynamic vehicle occlusion, as in frame 168 *Truck 2* starts to occlude *Truck 1*, which is completely shadowed in frame 200. The detector begins to see only the back part of the truck, until it loses it. Our tracker algorithm holds the vehicle’s target for 25 frames and after being unable to re-connect it, eliminates the target at frame 225. However, around frame 230, *Truck 1* appears again with a very different position in which only the front part is visible. Due to the lack of a vehicle model, the tracker is not able to associate it with the old target, but anyway is tracked and is confirmed as a new target in frame 270. At the end of the sequence, *Truck 1* stops at frame 405, the detector stops recognizing it as a dynamic object and the tracker keeps its target during one more second.

#### 4.1.2. Scene 2: AGV Turning Left at Intersection

**Figure 11:**
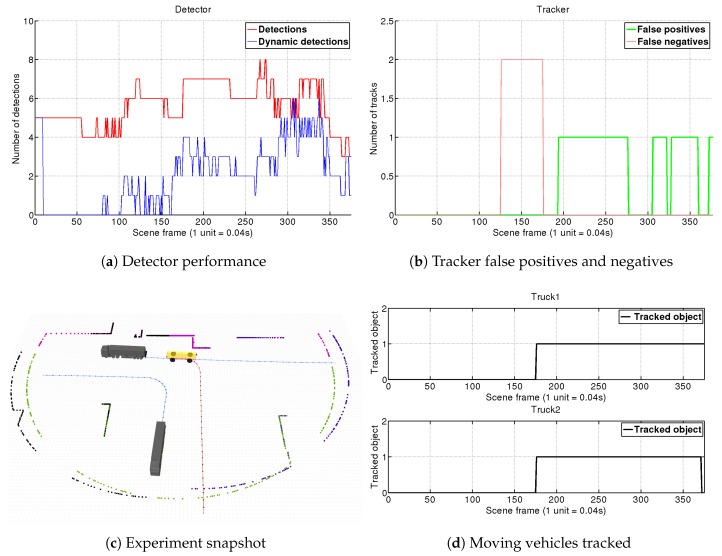
Scene 2. Our AGV approximates to an intersection for turning left. *Truck 2* comes from its left side and turns right. *Truck 1* comes from the right side and cross the intersection. When the intersection is free, the AGV turns left following *Truck 1*. Our system detects and tracks correctly both moving vehicles since they enter AGVs FOV until they stop or go out of range using only two LRFs.

In this simulation we use two LRFs sensors mounted on the front and the left side of the AGV. As seen in Figure 11c, the AGV approximates to an intersection for turning left. *Truck 1* is coming from the right side and cross the intersection. *Truck 2* comes from the left side and turns right to incorporate to the AGV’s road. When the intersection is free our vehicle turns left following *Truck 1*. Our main objective here is to test the algorithm capacities to group several detections from different sensors, which is a novelty introduced in this paper with respect to [12].

Results from this scene are shown in Figure 11. Trucks slowly enter the AGV’s FOV from frame 110 until frame 160. Since then, the detector filters between 4 and 5 objects as static (as seen in Figure 11a). It is not until frame 160 when the detector starts to be consistent on the dynamic detection, and therefore in frame 175 both objects are confirmed by the tracking. The simulation goes on and the system manages to track correctly both obstacles as shown in Figure 11d. At the end, in frame 370 *Truck 2* stops and is eliminated in frame 395. We can conclude that our system is able to correctly group and track different detections in a challenging situation with big changes of perspective and movements.

#### 4.1.3. Scene 3: AGV Turns Right at Intersection to Join Trucks Platoon

**Figure 12:**
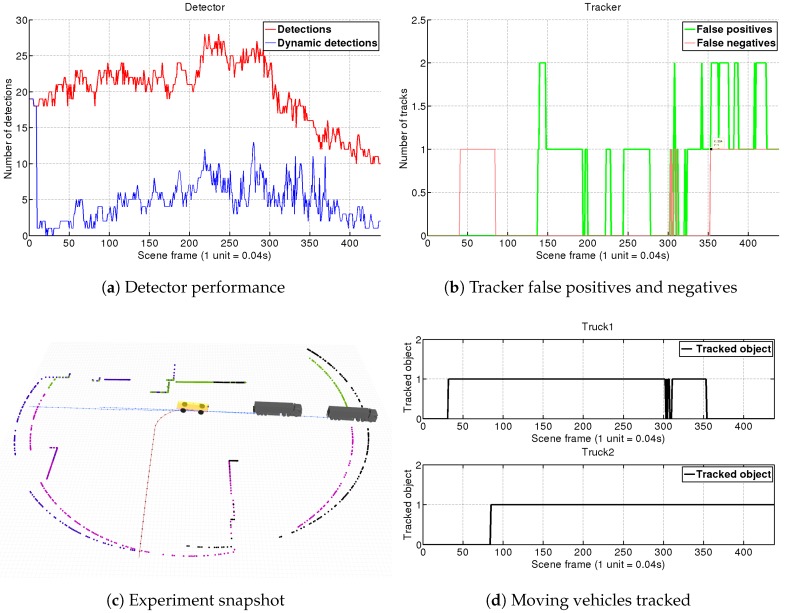
Scene 3. The AGV turns right to join a truck platoon, which is a situation of special interest in the autonomous transportation context. This simulation, performed with a FOV of $360^{\circ }$, shows the capacity of our DATMO system to quickly filter real moving obstacles from all the objects detected so that reducing by a quarter the computational burden of the tracker.

Platooning is one key feature that autonomous driving will introduce to make transportation more efficient. This scene aims to simulate our AGV joining a platoon of several vehicles after turning in an intersection, as can be observed in Figure 12c. We set four different LRF sensors on the vehicle obtaining $360^{\circ }$ of FOV. For each sensor, an object detector is instantiated, so this scenario will further test our grouping and filtering static obstacles capacities.

In the results shown in Figure 12, we can see how due to the increased FOV the number of objects detected rise to 28 elements peak. Analyzing Figure 12a we can conclude that our fast-matching algorithm is able to filter most of these detections leaving to the tracker just around a quarter of them. This is a big success, and represents an important reduction on the tracker computational load.

On the scene, it can be observed the 10-frame buffer for the initialization of the fast-matching module of the detector, in which all the segmented elements are considered as dynamics. *Truck 1* is already in the AGV’s FOV when the simulation begins, and it is confirmed by the tracker around frame 30, whereas *Truck 2* enters our FOV on frame 35 and is confirmed by the 85. We can observe how our performance is affected due to odometry drifts when the AGV turns right (which could be solved with better localization techniques). In this way, we observe an increase on the number of dynamic detections between frames 200 to 350 which generates some tracker false positives (see Figure 12b. Yet, as observed in Figure 12d, our system is robust enough and keeps track of both trucks, even of *Truck 1* when it gets almost lost due to occlusions from *Truck 2* during our turn around frame 300. When we are positioned in the platoon, *Truck 1* gets totally occluded and it is not tracked anymore from frame 350.

#### 4.1.4. Scene 4: AGV Witnesses a Truck Overtaking Another Truck

**Figure 13:**
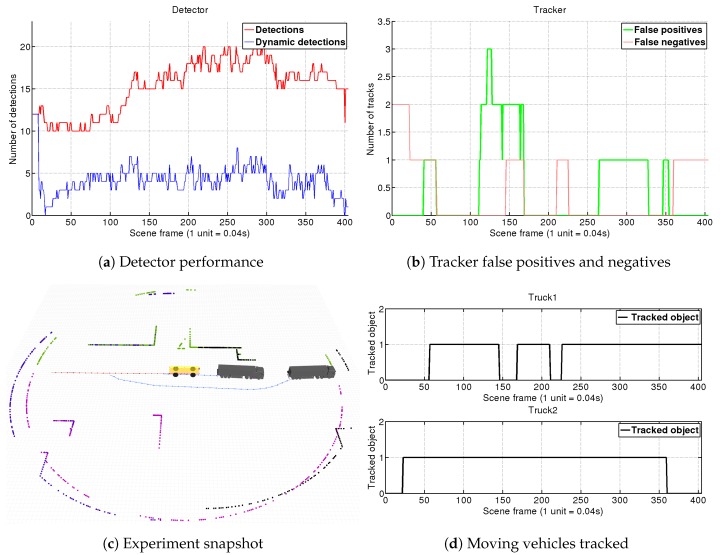
Scene 4. Taking part of a platoon, the AGV sees how the truck in front of it overtakes the head truck. It can be observed how in overtaking situations occluded vehicles become visible and the ones that were in sight get occluded, with the consequences that has to the DATMO system.

This simulation also uses four LRF sensors distributed around the AGV, and follows the previous line of platooning situations. As can be seen in Figure 13c, the AGV is situated at the back of two trucks and witnesses how *Truck 2* overtakes *Truck 1*. At this point, the AGV accelerates to cover the leaved gap and continues in the platoon formation.

In the results shown in Figure 13, we can again observe how the number of moving detections arriving to the tracker tagged as dynamic is two to four times lower than the number of total objects initially segmented by the four instantiated detectors. In the sequence, *Truck 2* starts its overtaking maneuver at frame 25. After this moment we will start to perceive *Truck 1* that was behind, which is confirmed by the tracker at frame 60. During the overtaking maneuver, both trucks are spatially close to each other and with similar speeds, which confuse our grouping strategy to group them at certain frames. Finally, at frame 330 when the maneuver of *Truck 2* ends, it gets occluded by *Truck 1* and therefore not detected or tracked.

### 4.2. Real Environment Experiment

**Figure 14:**
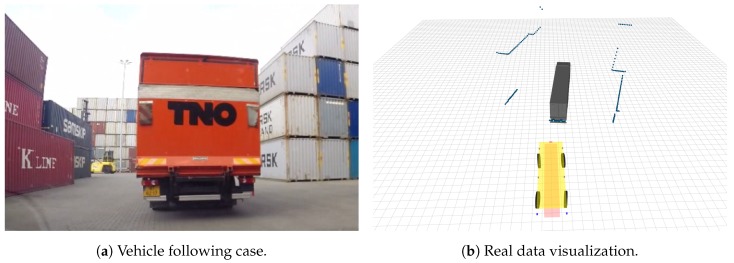
Real experimental sequence. Left, we show a snapshot of the sequence captured in the real port environment where the truck to be followed is appreciated. Right, we show the corresponding visualization of the 2D-LRF measurements obtained, along with the ground-truth of the vehicles.

After carefully validate our system over the simulated scenarios and find a balanced parameter configuration that is able to hold a good trade-off between precision vs recall and detection filtering vs tracking load, we evaluate our system in the same real scenario of [12] (see Figure 14), which was collected from the CTT port terminal of Hengelo, The Netherlands. Due to the scarce availability of real platforms as well as the need for several specific port authorizations to record the transport area premises we could only dispose of one complete real scene. In it, a sensorized Toyota Prius vehicle was acting as an AGV performing a vehicle following task. The vehicle was set with 6 Ibeo LUX laser scanners, mounted to capture pointcloud data over a 360 degrees FOV. Other sensors, which are detailed in [12] where available but not used here. Ibeo LUX laser scanners provide range measurements at four different layers covering a vertical FOV of 3.2 degrees and a horizontal FOV between 85 and 110 degrees with a resolution of up to 0.125 degrees. For our purposes, following our low-cost and minimum information objectives we only test our DATMO system using the third layer of the sensor, which was aligned with the horizon. We also use as additional input the longitudinal velocity of our own vehicle, integrating it to obtain an estimation of the ego-displacement through the experiment.

For autonomous vehicles, a high detection recall is of vital importance. As we were able to thoroughly calibrate our system in several scenarios in the simulated environment, we chose a parameter setup that, while keeps the system recall to the maximum, generalizes well in the different simulated and real scenarios working in real time.

Results of our presented approach are in the third column of Table 1. We also perform a comparison of these results against the ones obtained in [12]. In the previous work, we presented two different configurations, corresponding to the first two columns of the table. On the one hand, we presented a more restrictive detector configuration with higher matching thresholds, so that being stricter when tagging an object as dynamic. This configuration was linked to a tracker focused on keeping track of the generated targets because the detector recall was lower. On the other hand, we tuned a permissive detector with lower matching thresholds to obtain a high detection recall to not miss any possible dynamic object, with the drawback of introducing more false positive detections. To this configuration a tracker focused on filtering those false positives to get the real dynamic detections was attached. Due to the scarce data we had available, in [12] we tuned these two configurations using a subset of the real sequence that comprised the first few seconds. Results there were presented over the rest of the sequence. For absolute number comparisons, we include here in columns 1 and 2 of Table 1 the results of our initial DATMO approach from [12] although they are favored as contain the manually tuned frames. However, conclusions can be extracted about the performance of our updated modules.

Analyzing the results, we can extract several conclusions. Firstly, attending to the total number of segmented objects (first row), we can see how it gets decreased by our newly presented approach. After carefully reviewing the results, we can declare that this is due to the introduced dynamic threshold on the clustering step, as we now avoid over-segmentation of far objects. In addition, we can clearly observe the reduction on the number of objects tagged as *dynamic* obtained by the fast-matching step, as only part of the total segmented objects arrives at the tracker. In [12] this reduction factor was of 4.1 and 3.4 times respectively for the restrictive and permissive cases. Our current system shows a more permissive configuration with a 2.7 reduction factor (from 5232 to 1926). This permissive configuration can easily be explained from our generalization purposes, as we present a system that is able to properly work over a great variety of scenarios. Even though our tracker is still able to process the amount of inputs in more than real time, so this permissive configuration does not suppose any drawback for the system. Attending to the third row of Table 1, we can observe the capacities of our tracker to reduce the number of false positives. In the current approach, we can filter almost a 40% of the objects false positively tagged as dynamic, while obtaining a much higher system recall on the real sequence when compared to the previous versions. A video showing the real performance of our method can be found in https://youtu.be/El8mGBWESpI.

## 5. Conclusions

This work has presented a light and robust DATMO system that uses only the minimum information provided by 2D-LRFs. Our system aims to build on top of currently existing sensors of grid-based AGVs and only use single-layer laser rangefinders and odometry as input. Our approach divides the process in two stages, detection and tracking.

The detection algorithm, can segment any object in the port without any previous knowledge of its shape or model. Moreover, we perform a fast-matching object association between scans that consistently reduces between two and five times the number of elements that arrive at the tracker by selecting the ones with strong dynamic cues.

An improved version of MHT has been presented allowing us to track the dynamic objects. We complemented the MHT by defining special functions to confirm, hold or delete targets. Moreover, we have defined velocity filters to discard static objects in local coordinates as well as grouping approaches that are able to track jointly different detections of the same object.

The system has been evaluated both in simulated and real data obtained in the CTT port terminal in Hengelo, the Netherlands. The results show that, although very little information is provided by one single-layer laser scanners, our DATMO system accomplish perfectly different common situations for autonomous transportation such as following vehicles, joining platoons or observing traffic approaching to an intersection.

## Figures and Tables

**Table 1:** Results over the real captured sequence of the presented extended DATMO system.

	Restrictive Case [12]	Permissive Case [12]	Ours
Total Segmented Obj.	5345	5393	5232
Total Dynamic Det.	1287	1557	1926
Total Tracks	1069	1085	1159
System Recall (%)	0.8842	0.9353	0.9816

## Abbreviations

**Acronyms**	
DATMO	Detecting and Tracking of Moving Objects
LRF	Laser RangeFinder
AGV	Autonomous Guided Vehicle
MHT	Multiple-Hypotheses Tracking
ACT	Automated Container Terminal
FLIRT	Fast Laser Interest Region Transform
SIFT	Scale-invariant feature transform
SURF	Speeded-Up Robust Features
MDP	Markov Decision Process
KF	Kalman Filter
JPDAF	Joint Probabilistic Data Association Filtering
MH-KF	Multiple-Hypothesis Tracking based on Kalman Filters
PDBS	Point-Distance-Based Segmentation
PCA	Principal Component Analysis
FOV	Field of View
	
**Variables**	
$Sc_{t}$	Laser Scan in time *t*
$r_{i}$	Range measurement
$\alpha_{i}$	Bearing angle
S	Set of point on a scan
*k*	Number of scans buffered
$\cdot^{veh}$	Frame referred to the vehicle
$\ell_{1}\;;\;\ell_{2}$	Absolute/Euclidean Distance
$Th_{\left(\cdot \right)}$	Threshold
$obj_{j}$	Observed Object
J	Set of segmented objects
$ref_{act}$	Current reference frame of an object
wth;lth	Object’s width/length
φ	Object’s orientation regarding the longitudinal axis
np	Number of laser points in an object
L	Set of extracted lines in an object
$pt_{\left(\cdot \right)}$	Laser Point in Euclidean coordinates
ϵ	Regression error of the line
$\vec{\theta }$	Director vector for the line
np	Number of laser points of and object
C	Set of extracted corners in an object
ρ	Corner’s orientation
γ	Corner’s aperture
P	Perimeter
A	Area
Tg	Tracked Dynamic Target
Z	Dynamic observations
$M_{t}$	Set of dynamic detections at time *t*
x	Tracked Targets
N	Set of Targets tracked at time *t*
H	Measurement matrix
$\bar{x}$	Propagation of the target state
$\bar{P}$	Covariance matrix of the propagated target
R	Covariance matrix of the detection
η	Target association threshold
${P_{i}}^{t}$	Probability of the actual hypothesis
$\beta_{fal}$	Poisson probability distribution for false alarms
$\beta_{new}$	Poisson probability distribution for new targets
$N_{fal}$	Number of detections associated with false alarms
$N_{new}$	Number of detections associated with new targets
$P_{det}$	Probability of detection
$N_{det}$	Number of detections associated with existing targets
$N_{TGT}$	Number of the existing targets
$N_{detector}\left(Z_{m}-H\bar{x},B\right)$	Gaussian probability distribution of the detections for the detector
$P_{nc}$	Probability distribution for the not confirmed targets
$N_{nc}$	Number of times that the target has a detection associated with it
$P_{h}$	Probability distribution to hold a dynamic target
$N_{na}$	Number of targets without dynamic detection associated
${\vec{V}}_{obj}$	Object velocity
${\vec{V}}_{veh}\;;\;{\vec{W}}_{veh}$	Ego vehicle linear/angular velocity
$\vec{d}$	Distance between ego vehicle and tracked object
λ	Weight controlling the rise of confirmation probability
